# Refusal of treatment among HER2-positive breast cancer patients in China: a retrospective analysis

**DOI:** 10.3389/fpubh.2023.1305544

**Published:** 2024-01-18

**Authors:** Xin Wang, Zhiwei Lian, Qiyou Wu, Fan Wu, Gong Zhang, Jian Liu, Chuanben Chen, Jing Sun

**Affiliations:** ^1^School of Health Policy and Management, Chinese Academy of Medical Sciences and Peking Union Medical College, Beijing, China; ^2^Clinical Oncology School of Fujian Medical University, Fujian Cancer Hospital, Fuzhou, China

**Keywords:** breast cancer, human epidermal growth factor receptor 2, targeted therapy, treatment refusal, binary logistic regression

## Abstract

**Background:**

There is a need to update the understanding of treatment refusal among cancer patients in China, taking into account recent developments. This study investigated how public insurance coverage of the first breast cancer targeted therapy contributed to the changes in treatment refusal among HER2-positive breast cancer patients in China. And it intensively examined and discussed additional barriers affecting patient utilization of innovative anticancer medicines based on the types and reasons for treatment refusal.

**Methods:**

This retrospective study included female breast cancer patients diagnosed as HER2-positive who received treatment at a provincial oncology center in southern China between 2014 and 2020. Multivariable analysis was conducted using a binary logistic regression model. Subgroup analysis was performed with the same regression model.

**Results:**

Among the 1,322 HER2-positive breast cancer patients who received treatment at the study hospital between 2014 and 2020, 327 (24.55%) had ever refused treatment. Economic reasons were reported as the primary cause by 142 patients (43.43%). Patients diagnosed after September 2017, when the first breast cancer targeted therapy was included in the public health insurance, were less likely to refuse treatment (OR = 0.64, 95% CI:0.45 ~ 0.91, *p* = 0.01) compared to those diagnosed before September 2017. Patients enrolled in the resident health insurance were more likely to refuse treatment (OR = 2.43, 95% CI:1.77 ~ 3.35, *p* < 0.001) than those enrolled in the employee health insurance.

**Conclusion:**

This study reveals a high rate of treatment refusal among HER2-positive breast cancer patients, primarily attributed to financial factors. The disparity in public health insurance benefits resulted in a heavier economic burden for patients with less comprehensive benefits. Furthermore, the study identified challenges faced by patients seeking quality-assured cancer care in underdeveloped regions in China. By addressing economic barriers, promoting accurate health information, and improving cancer care capacity across the country can reduce the rate of treatment refusal.

## 1 Introduction

Breast cancer has shown increasing incidence and mortality rates in China, making it the most prevalent cancer among women (1–3). Human epidermal growth factor receptor-2 (HER-2) positive breast cancer is characterized by its aggressive nature, rapid progression, resistance to conventional chemotherapy and poor prognosis (4, 5). The prognosis of this challenging disease has been improved through the introduction of targeted therapy, which has demonstrated superior efficiency compared to traditional chemotherapy and endocrine therapy (6, 7). The advancement in treatment technology also led to improvements in the 5-year survival rate of breast cancer patients to 83.2% in China. However, there is still room for improvement when comparing the survival rate in China with the United States (90.2%) and Japan (89.4%) (2, 8).

Existing studies have shown that treatment adherence is a critical factor affecting cancer survival (9). And treatment adherence varies among patients. Apart from demographic and clinical characteristics of age, hormone levels and cancer stage, social and economic factors such as income and level of health care coverage are also associated with treatment adherence (10–24). To further raise the survival rate of HER-2 positive breast cancer, it is critical to reduce the number of treatment refusal, and to promote active engagement of patients in sustained and standardized treatment. Patients facing financial difficulties are more likely to refuse targeted therapy due to the substantial financial burden it imposes. The inclusion of the first breast cancer targeted therapy in China’s public health insurance in September 2017 marked a significant milestone in mitigating the financial burden experienced by patients (25–27). It is important to know if this policy led any changes of the refusal rate.

International studies have extensively examined treatment refusal among cancer patients. Majority of studies conducted in China focused on treatment refusal among cancer patients before the end of 2017, which revealed 40–55% refusal rate, higher than that in other countries or regions (28–30). There is a need to update the understanding of treatment refusal among cancer patients in China, taking into account of the recent developments, and to verify if the national health insurance coverage of targeted therapies brought any changes of the refusal rate. Therefore, the primary objective of this study is to investigate how public health insurance coverage of the first breast cancer targeted therapy contributed to the changes in the refusal of physician-recommended treatment among HER2-positive breast cancer patients and identify other current barriers to patient access to innovative medicines. The findings of this study will provide evidence for decision-makers to enhance treatment adherence and improve public health insurance coverage for innovative anticancer medicines.

## 2 Methods

### 2.1 Data source

**Figure 1:**
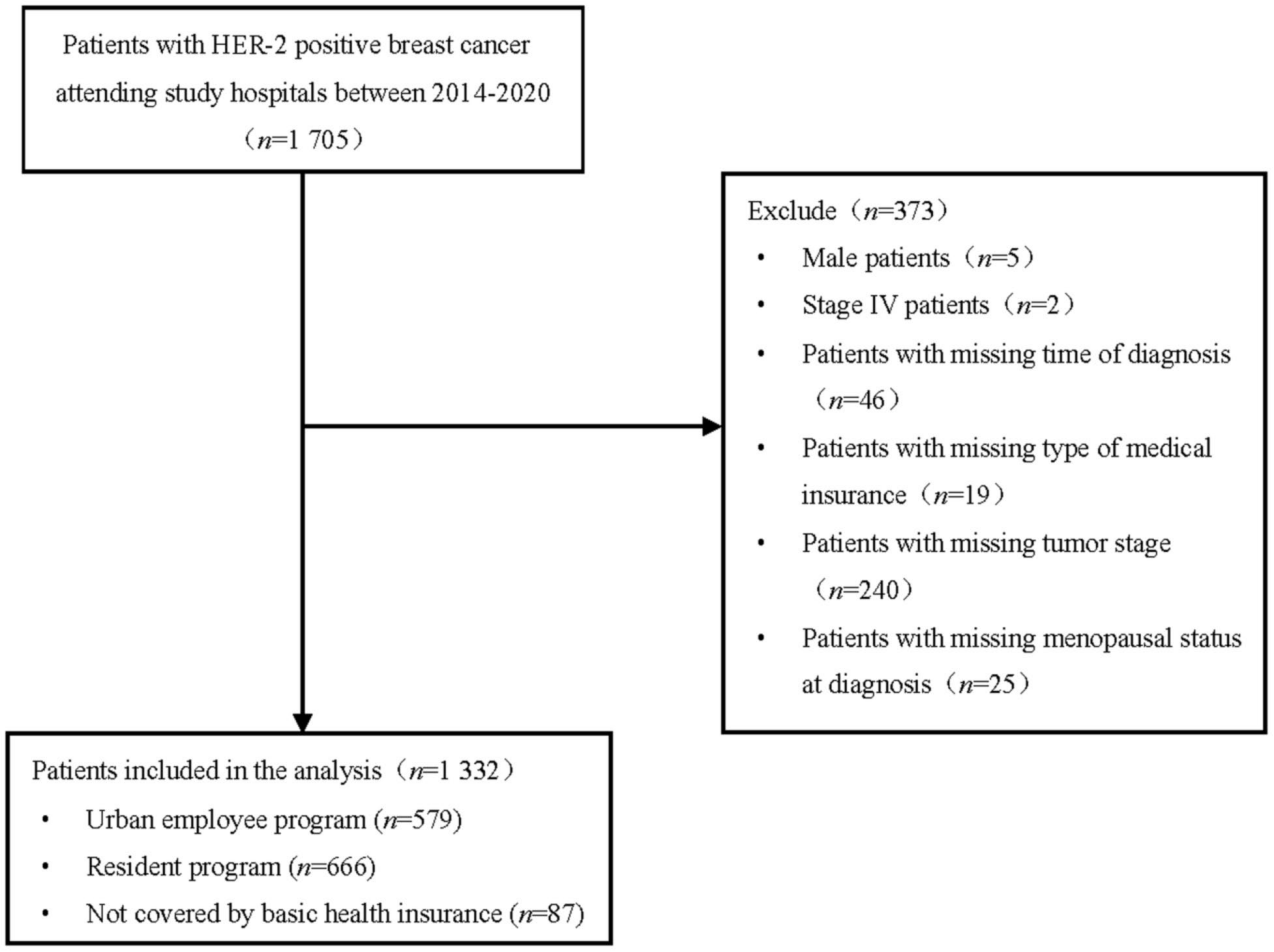
Flow chart of sample selection.

This is a single-center study at the provincial oncology center of Fujian province, located in southern China with a median level of economic development and number of population, which is more representative than other provinces. The study population consisted of female breast cancer patients who visited the study hospital between 2014 and 2020, and were diagnosed as HER 2-positive (Figure 1). The inclusion criteria of participants were as follows: (1) female; (2) histological staging of invasive breast cancer and a confirmed diagnosis of HER2-positive gene target, based on the national guidelines for diagnosis and treatment of breast cancer (31); and (3) availability of medical records at the study hospital from 2014 to 2020. Patients were excluded if they met any of the following criteria: (1) missing variables required for the analysis, (2) male breast cancer, or (3) diagnosed with stage IV breast cancer, or (3) patients with a history of other malignant tumors or comorbidity. Given that the treatment plan and refusal behavior of stage IV patients are significantly different from that of patients diagnosed as stage I-III, and there were only two stage IV cases, they were excluded.

### 2.2 Data collection

Two trained oncology physicians working in the study hospital helped to extract the refusal information independently in parallel. Considering that there was an upgrade and significant change of the hospital health information system in 2013, treatment refusal records between 2014 and 2020 of all the included patients from the unstructured electronic medical records were retrieved by searching the key word of “refusal.” Documentation of any recommended therapy declined by either the patients or their family members or guardian, were considered to have a refusal of treatment. Related records of the refusals including patient ID, time, type of treatment, reasons of refusal were extracted. If the two physicians had a disclaimer on whether the patient refused treatment, they revisited the medical record together to verify and confirm. The scope of refused treatments included surgery, chemotherapy, radiotherapy, targeted and hormone therapy. Demographic and sociological characteristics of the included patients were obtained from the structured electronic medical records.

### 2.3 Study design

We conducted a case–control retrospective analysis to examine how public health insurance coverage of the first breast cancer targeted therapy contributed to changes in treatment refusal among HER2-positive breast cancer patients. The participants were stratified into two groups based on tumor diagnostic stage: early- (stage I, II) and advanced-stage (stage III). Subgroup analysis was conducted within these two groups.

### 2.4 Variables

The primary outcome variable of this study was patients’ refusal of treatment recommended by their physicians, `which was defined as a patient having refused a treatment recommendation given by the attending doctor, including surgery, chemotherapy, radiotherapy, targeted and hormone therapy, in the patient’s medical record. During the study period, patients who had ever refused recommended treatment was denoted as 1, while those who had never refused recommended treatment was denoted as 0. The independent variable was the time of diagnosis. We chose the time of diagnosis to consider the economic impact of public protection policies on patient care in 2017. Diagnosed time before the end of 2017 was assigned a value of 1, and after 2017 was assigned a value of 0. Several potential factors that could influence treatment refusal were selected based on professional judgment by previous studies and oncologists. These factors encompassed the variables of patient age (categorised in “<40”; “40–59”; and “≥60”) (10, 12–14, 16–24), type of public health insurance (categorised in “Urban employee program”; “Resident program”; and “Not covered by basic health insurance”) (10, 12–14, 17–20, 22, 24), treatment place (“Not Local resident” =0; “Local resident” = 1) (10, 14, 17, 23, 24), stage at diagnosis (“I, II” = 0; “III” = 1; based on the American Joint Committee on Cancer classification) (10, 12, 13, 15, 18, 19, 21, 23, 24), menopausal status at diagnosis (31) (“patient is not menopausal at the time of diagnosis” = 0; “patient is menopausal at the time of diagnosis” = 1), estrogen receptor expression (24) (“Negative” = 0; “Positive” = 1), and progesterone receptor expression (24) (“Negative” = 0; “Positive” = 1).

### 2.5 Statistical analysis

Statistical analysis was conducted using SPSS 26.0 software. Descriptive analyzes were performed for demographic, socioeconomic and clinical factors and refusal reasons. All the demographic and clinical characteristic variables were categorical variables. The differences in the distribution of these characteristics between the refusal group and the non-refusal group were assessed using the *χ*^2^ test. Logistic multiple regression model was employed to investigate whether public health insurance coverage of the first breast cancer targeted therapy contributed to any change in treatment refusal among HER2-positive breast cancer patients. The model included the identified potential factors as control variables, including time of diagnosis, age at diagnosis, diagnosed tumor stage, type of basic health insurance coverage, local resident or not, and hormone levels. Despite the wide use of setting a defined value of p for selection of candidate predictors in the univariate regression analysis, considering that the selection is sometimes not good for fitting the model in line with the practical situations, and we have a relatively large sample size, we did not include candidate predictors based on a set *p*-value. Instead, we included all of the above variables in the logistic multiple regression. To test the robustness of the findings, subgroup regression analysis was conducted for patients with early tumor diagnosis stages (stage I, II) and advanced tumor stages (stage III). All models were fitted using the entry method (32). The significance level was set at *α* = 0.05.

## 3 Results

### 3.1 Patient characteristics

Table 1 presents the characteristics of the 1,332 female patients diagnosed with HER2 positive breast cancer between 2014 and 2020. Among them, 327 patients (24.55%) had documented treatment refusal; 909 were diagnosed prior to the end of 2017, when the first breast cancer targeted therapy was included in the national public health insurance through price negotiation; 579 patients (43.47%) were insured under urban employee health insurance, while 666 patients (50.00%) were under resident health insurance; and 827 patients (62.09%) were diagnosed with early-stage tumors (stage I, II) at the time of diagnosis. Among the 1,322 patients, 780 (58.56%) were non- local residents.

**Table 1:** Summary of the 1,332 sample patients.

Characteristics	All patients (*n* = 1,332)		Patients experienced treatment refusal (*n* = 327)		Patients not experienced treatment refusal (*n* = 1,005)		*p* value
	No. of patients	%	No. of patients	%	No. of patients	%	
*Diagnosed time (Targeted therapy started to be included in public health insurance)*							0.68
Before end of 2017	909	68.24	220	67.28	689	68.56	
After 2017	423	31.76	107	32.72	316	31.44	
*Diagnosed tumor stage*							**0.001**
I, II	827	62.09	177	54.13	650	64.68	
III	505	37.91	150	45.87	355	35.32	
*Age at diagnosis*							0.64
<40	219	16.44	59	18.04	160	15.92	
40 ~ 59	969	72.75	232	70.95	737	73.33	
≥60	144	10.81	36	11.01	108	10.75	
*Type of basic health insurance coverage*							**<0.001**
Urban employee program	579	43.47	106	32.42	473	47.06	
Resident program	666	50.00	201	61.47	465	46.27	
Not covered by basic health insurance	87	6.53	20	6.12	67	6.67	
*Local resident or not*							0.75
Yes	552	41.44	138	42.20	414	41.19	
No	780	58.56	189	57.80	591	58.81	
*Menopause or not at the time of diagnosis*							0.48
Yes	587	44.07	150	45.87	437	43.48	
No	745	55.93	177	54.13	568	56.52	
*Estrogen receptor (ER)*							0.07
Positive	716	53.75	190	58.10	526	52.34	
Negative	616	46.25	137	41.90	479	47.66	
*Progestogen receptor (PR)*							0.09
Positive	569	42.72	153	46.79	416	41.39	
Negative	763	57.28	174	53.21	589	58.61	

Bold values indicate statistically significant.

Among the 909 patients diagnosed before the end of 2017 and the 423 patients diagnosed after 2017, 220 (24.20%) and 107 (25.30%) patients respectively, had experienced treatment refusal. The difference in the percentages of patients who experienced treatment refusal between these two periods was not statistically significant. There were statistically significant differences in the distributions of public health insurance enrolments (*p* < 0.001) and tumor diagnosis stages (*p* = 0.001) between the patients who experienced treatment refusal and those who did not experience treatment refusal. A higher proportion of patients insured under resident health insurance (61.47%) had experienced treatment refusal compared to all 1,332 patients included in the study (50.00%) and patients who did not experience treatment refusal (46.27%). Among the 579 patients insured under urban employee health insurance and the 666 patients insured under resident health insurance, there were 106 (30.18%) and 201 (18.31%) cases of treatment refusal, respectively.

### 3.2 Reasons and types of treatment refusal

Table 2 provides further details of the 327 patients who experienced treatment refusal. Among the 327 patients, 220 (67.28%) were diagnosed before the end of 2017, while 107 (32.72%) were diagnosed after 2017. A total of 201 patients (61.47%) were enrolled in the resident health insurance. The distribution of health insurance coverage and tumor diagnosis stages among the 327 patients who refused treatment was similar to that of the 206 patients who refused targeted therapy. Among the subgroups categorized by time of diagnosis, type of health insurance coverage and tumor diagnosis stages, at least 60% of patients in each subgroup refused targeted therapy. Examining the 142 patients who refused treatment due to financial reasons, 88 (62%) were diagnosed before the end of 2017, 54 (38%) were diagnosed after 2017, and the majority (92 accounted 64.8%) were insured under resident health insurance.

**Table 2:** Summary of 327 patients who experienced treatment refusal.

Characteristics of patients	Diagnosed time *n* (%)		Type of basic health insurance coverage *n* (%)			Local resident *n* (%)		Diagnosed tumor stage *n* (%)		Total
	Before end of 2017	After 2017	Urban employee program	Resident program	Not covered by basic health insurance	Yes	No	I, II	III	
*Types of treatment refusal*										
Targeted therapy	132 (64.10%)	74 (35.90%)	64 (31.10%)	130 (63.10%)	12 (5.80%)	90 (43.69%)	116 (56.31%)	111 (53.90%)	95 (46.10%)	206 (63.00%)
Other therapies	88 (72.70%)	33 (27.30%)	42 (34.70%)	71 (58.70%)	8 (6.60%)	48 (39.67%)	73 (60.33%)	66 (54.50%)	55 (45.50%)	121 (37.00%)
*Reasons of treatment refusal*										
Economic reason	88 (62.00%)	54 (38.00%)	39 (27.50%)	92 (64.80%)	11 (7.70%)	61 (42.96%)	81 (57.04%)	74 (52.10%)	68 (47.90%)	142 (43.43%)
Shift to traditional Chinese Medicine treatment	4 (80.00%)	1 (20.00%)	2 (40.00%)	3 (60.00%)	0 (0.00%)	3 (60.00%)	2 (40.00%)	1 (20.00%)	4 (80.00%)	5 (1.53%)
Shift to treatment at local hospital	2 (40.00%)	3 (60.00%)	0 (0.00%)	5 (100%)	0 (0.00%)	0 (0.00%)	5 (100%)	4 (80.00%)	1 (20.00%)	5 (1.53%)
Due to adverse drug reactions	17 (89.50%)	2 (10.50%)	12 (63.20%)	6 (31.60%)	1 (5.30%)	13 (68.43%)	6 (31.57%)	18 (94.70%)	1 (5.30%)	19 (5.81%)
Discontinue treatment without any further medical intervention	2 (50.00%)	2 (50.00%)	2 (50.00%)	1 (25.00%)	1 (25.00%)	1 (25.00%)	3 (75.00%)	2 (50.00%)	2 (50.00%)	4 (1.22%)
Unknown reasons	107 (70.40%)	45 (29.60%)	51 (33.60%)	94 (61.80%)	7 (4.60%)	60 (39.48%)	92 (60.52%)	78 (51.30%)	74 (48.70%)	152 (46.48%)
*Total*	220 (67.28%)	107 (32.72%)	106 (32.41%)	201 (61.47%)	20 (6.12%)	138 (42.21%)	189 (57.79%)	177 (54.13%)	150 (45.87%)	327 (100%)

As summarized in Table 3, out of the 327 patients who experienced treatment refusal, economic factors were the main reasons for refusal in 142 cases (43.43%). Among the 142 patients, 131 refused targeted therapy. Within the larger group of 327 patients, 206 (63.00%) refused targeted therapy, with financial constrains being the primary factors for 131 out of 206 (63.59%), 88 (62.00%) were diagnosed before end of 2017, 92 (64.8%) were covered by resident health insurance, 81 (57.04%) were non-local residents. Five out of the 327 patients refused the treatment recommended by their doctors and opted for traditional Chinese medicine (TCM) treatment, among whom, four were diagnosed before end of 2017, three were resident health insurance enrollees, four had advanced-stage (stage III) tumors (Table 2), three refused targeted therapy (Table 3). Five out of the 327 patients requested to shift to treatment at local hospital, all of whom were covered by the resident health insurance, and four were in the early tumor stages (stage I and II) (Table 2). Adverse drug reactions (ADRs) led to treatment refusal in 19 out of the 327 patients. Among the 19 cases, 17 were patients diagnosed before end of 2017, two patients refused targeted therapy, 17 patients refused other therapies (Table 3), majority of which were chemotherapies (15 cases). Moreover, four out of the 327 patients requested to discontinue treatment without any further medical intervention. Among the four cases, three patients were undergoing treatment outside their residential areas (Table 2).

**Table 3:** Reasons and types of treatment refusal of 327 patients who experienced treatment refusal.

Reason of treatment refusal	Types of treatment refusal *n* (%)		
	Targeted therapy	Other therapies	Total
Economic reason	131 (40.06%)	11 (3.36%)	142 (43.43%)
Shift to traditional Chinese medicine treatment	3 (0.92%)	2 (0.61%)	5 (1.53%)
Shift to treatment at local hospital	1 (0.31%)	4 (1.22%)	5 (1.53%)
Due to adverse drug reactions	2 (0.61%)	17 (5.20%)	19 (5.81%)
Discontinue treatment without any further medical intervention	0 (0.00%)	4 (1.22%)	4 (1.22%)
Unknown reasons	69 (21.10%)	83 (25.38%)	152 (46.48%)
Total	206 (63.00%)	121 (37.00%)	327 (100%)

### 3.3 Factors associated with treatment refusal

The results of the multivariable logistic regression analysis are presented in Table 4. Among the 1,332 patients included in this study, by controlling for other variables, it was found that patients diagnosed after 2017 were less likely to refuse treatment compared to those diagnosed before the end of 2017 (OR = 0.64, 95% CI:0.45 to 0.91, *p* = 0.01). This finding was consistent with the result obtained from the subgroup analysis among patients with early-stage tumor diagnosis (stage I, II) (52%, OR = 0.52, 95% CI:0.32–0.85, *p* = 0.01). It was also observed that, patients enrolled in the resident health insurance were more likely to refuse treatment compared to those enrolled in the employee health insurance (OR = 2.43, 95% CI:1.77 to 3.35, *p* < 0.001). This finding is consistent with the results obtained from the subgroup analyzes among patients with early-stage (stage I, II) (OR = 2.37, 95% CI:1.55–3.63, *p* < 0.001) and advanced-stage (stage III) tumors (OR = 2.60, 95% CI:1.60–4.23, *p* < 0.001).

**Table 4:** Logistic regression analysis of predictors for treatment refusal among all included 1,332 patients and subgroups.

Characteristics	All patients			Tumor stage I, II			Tumor stage III		
	OR	95% CI	*p* value	OR	95% CI	*p* value	OR	95% CI	*p* value
Diagnosed time									
Before end of 2017 (ref.)									
After 2017	0.64	0.45 ~ 0.91	**0.01**	0.52	0.32 ~ 0.85	**0.01**	0.80	0.48 ~ 1.36	0.42
*Diagnosed tumor stage*									
I, II (ref.)									
III	1.55	1.2 ~ 2.01	**0.001**						
*Diagnosis age*									
<40 (ref.)									
40 ~ 59	0.91	0.63 ~ 1.32	0.63	0.86	0.52 ~ 1.4	0.53	1.01	0.57 ~ 1.79	0.97
≥60	1.15	0.64 ~ 2.07	0.65	1.08	0.49 ~ 2.38	0.84	1.28	0.52 ~ 3.17	0.59
*Type of basic health insurance coverage*									
Urban employee program (ref.)									
Resident program	2.43	1.77 ~ 3.35	**<0.001**	2.37	1.55 ~ 3.63	**<0.001**	2.60	1.6 ~ 4.23	**<0.001**
Not covered	1.41	0.81 ~ 2.44	0.22	1.78	0.9 ~ 3.5	0.10	0.93	0.36 ~ 2.41	0.87
*Local resident*									
Yes	1.15	0.88 ~ 1.49	0.31	1.09	0.76 ~ 1.55	0.64	1.22	0.82 ~ 1.82	0.33
No (ref.)									
*Menopause*									
Yes	1.21	0.91 ~ 1.6	0.19	1.36	0.94 ~ 1.98	0.11	1.05	0.68 ~ 1.62	0.83
No (ref.)									
*Estrogen receptor (ER)*									
Positive	1.20	0.85 ~ 1.7	0.29	1.47	0.93 ~ 2.32	0.10	0.89	0.52 ~ 1.51	0.67
Negative (ref.)									
*Progestogen receptor (PR)*									
Positive	1.14	0.81 ~ 1.62	0.46	0.98	0.62 ~ 1.55	0.93	1.39	0.81 ~ 2.39	0.23
Negative (ref.)									

Bold values indicate statistically significant.

## 4 Discussion

This study examined a total of 1,332 HER2-positive breast cancer patients diagnosed at a provincial oncology center in southern China between 2014 and 2020, and revealed that 24.55% of patients had ever refused treatment. Although this proportion is lower than the results reported in previous domestic studies conducted before 2018, it remains higher than the reported proportion of treatment refusal among cancer patients in high-income countries. A study conducted in another provincial oncology center in southern China in 2013 reported that, among 386 colorectal cancer patients, 41.5% refused adjuvant chemotherapy (30). Another study conducted in a provincial hospital in southern China found that 43.8% of 2,794 lung cancer patients refused anticancer treatment, and this proportion showed no decreasing trend from 2013 to 2017 (29). In comparison, studies conducted in high-income countries have shown comparatively lower rates of treatment refusal. For instance, a study conducted in the United States on head and neck cancer patients between 2004 and 2014 found that only 1.3% of the 233,389 registered patients had ever refused treatment, including surgery, radiation therapy, or chemotherapy (10). Similarly, a retrospective cohort study conducted in the United States between 2004 and 2016 on invasive breast cancer cases reported that 14.98% of the 2,058,568 patients had ever refused treatment. The specific refusal rates were 6% for surgery, 14.1% for chemotherapy, 5.5% for radiation and 6.3% for endocrine therapy (17). Other studies conducted in the United States between 2017 and 2021 have reported refusal percentages ranging from 1 to 14.98% for different types of cancer treatment (10, 17, 33–37). In Canada, a study conducted on 15,427 breast cancer patients in the Northern Alberta Health Region from 1980 to 2006 reported that only 1.2% of patients refused the standard primary treatment (38). Similarly, a study conducted at the Nottingham Breast Institute in the United Kingdom, which included 268 female patients over 70 years of age diagnosed with early operable primary breast cancer (<5 cm), reported a treatment refusal rate of 1.5% (39). A systematic review and meta-analysis revealed that abandonment rates (ARs) in patients with leukemia were significantly higher in lower-middle-income countries compared to upper-middle-income countries (UMICs) (29% versus 2%). Notably, China had the highest ARs (34%) among the UMICs included in the study (40).

An important finding of this study is that economic factors emerged as the primary reason for treatment refusal among HER2-positive breast cancer patients in China, with financial considerations being particularly prominent in the refusal of targeted therapies. This finding aligns with the result of our logistic multivariable regression analysis, which indicated a lower likelihood of treatment refusal after the first breast cancer targeted therapy was included in the health insurance. Additionally, our regression analysis revealed that patients enrolled in the resident health insurance with lower benefits were more likely to refuse treatment compared to those enrolled in the employee health insurance with better benefits. In September 2017, China made significant progress by including the first breast cancer targeted therapy in health insurance through price negotiation, resulting in a substantial reduction in price (27). The price of that targeted therapy decreased from approximately USD 3500 to USD 1100 per dose. Subsequent insurance coverage contract renewals further decreased the price to USD 800 per dose (41). By the end of 2020, a total of four breast cancer targeted therapies had been included in the national health insurance (42). The inclusion of targeted therapies in health insurance has significantly alleviated the financial burden for breast cancer patients, although out-of-pocket expenses for targeted therapies remains higher compared to conventional treatments. Therefore, patients receiving targeted therapies still face higher economic burdens. Consequently, resident health insurance enrollees entitled to less comprehensive benefits were more likely to refuse treatment compared to the employee health insurance enrollees with better benefits.

The issue of cancer patients refusing treatment due to economic factors is not unique in China, but also exists in the United States, a country with advanced healthcare but without universal health coverage. A study found that among 531,700 registered patients from 2004 to 2013, lack of health insurance was identified as a risk factor associated with treatment refusal (12). Similarly, another study conducted from 2007 to 2015 on 318,318 cancer patients in the United States revealed that uninsured individuals or Medicaid beneficiaries were significantly more likely to refuse treatment (13). Although China has achieved universal coverage of basic medical insurance, disparities in insurance coverage still exist among different groups of insured individuals. The benefits of employee health insurance are significantly higher than those of resident health insurance, contributing to the economic burden faced by patients enrolled in resident health insurance. In 2020, the average annual disposable income *per capita* was approximately USD 6200 for urban residents and only USD 2500 for rural residents in China (43). Research suggests that after targeted cancer therapies were included in the health insurance, the direct medical expenses for breast cancer patients who completed at least one standard course of targeted treatment, or maintained treatment until disease progressed amounted to approximately USD 26,500 (2017–2019). A total of 49.03% of the expenditures were out-of-pocket (OOP) payments (44, 45). Even with better health insurance benefits (with a deductible of USD 260, 10% OOP payment before insurance reimbursement, and a 70% reimbursement rate), it was estimated that Chinese urban and rural residents would need to spend 1.55 and 3.97 years of their total income, respectively, to afford the afore mentioned treatment expenses. Individuals with lower health insurance benefits face a greater economic burden when receiving the same treatment. To ensure that economic factors do not hinder access to necessary treatments, it is crucial to provide adequate financial protection and to reduce OOP expenses for cancer treatments. By addressing these issues, more patients can benefit from the inclusion of innovative anticancer medicines in health insurance and have improved access to necessary treatments, ultimately contributing to better overall cancer outcomes.

In addition to economic factors, this study identified patient concerns about ADRs as a contributing factor to treatment refusal, particularly in the context of traditional chemotherapy. Similar reasons have been reported in some developing countries. For example, a study conducted in Indonesia involving interviews with healthcare professionals confirmed that patients often abandon treatment due to fear of medication toxicity and ADRs (46). The proportion of treatment refusal due to ADRs is relatively lower in targeted therapy compared to traditional chemotherapy. This can be attributed to the high specificity and low toxicity associated with targeted therapy. This study also revealed that a small number of patients refused the treatment recommended by their doctors and instead opted for TCM treatment. The preference for TCM among Chinese cancer patients can be attributed to the belief that TCM enhances physical fitness, improves overall health and reduces the side effects of conventional treatment (47). The utilization of TCM among cancer patients in China is relatively common. A study conducted in 35 general hospitals in central China reported that 72.24% of cancer patients incorporated TCM into their cancer treatments (48). Similarly, a telephone survey conducted on colorectal cancer patients in China found that out of 160 patients who refused chemotherapy, 10% did so because they trusted and chose TCM treatment (30). TCM, as a form of complementary therapy, may have positive effects on regulating the overall immune system and gastrointestinal function of cancer patients, especially for those undergoing chemotherapy or radiotherapy and experiencing ADRs. However, there is currently no scientific evidence supporting the use of TCM as a monotherapy for improving cancer survival and prognosis. Oncologists must clearly explain the risks associated with relying solely on TCM treatment and rejecting standard therapies recommended by the national treatment guidelines. It is essential to enhance the dissemination of scientific health information to the public to ensure a correct understanding of TCM.

Furthermore, this study has also identified patients’ preference to receive treatment in their hometown as a significant reason for treatment refusal, particularly among patients who seek cancer treatment outside of their residential areas. By reviewing the demographic and social characteristics of the four patients who cease treatment due to family matters, we found that three of them were not local patients. Non-local patients were more likely to cease treatment. The primary factor driving this preference is the inconvenience and increased financial burden associated with frequent travels between their hometown and the distant oncology center located in the capital city of the province. This finding highlights that specialized oncology services in certain regions of China may be limited, making it difficult to meet the basic healthcare needs of cancer patients. Consequently, patients from these regions are compelled to travel far from their residential areas to seek oncology treatment. Previous studies also highlighted that the distance to healthcare facilities significantly limits the accessibility of medical services (49). To address this issue, it is imperative to enhance the capabilities and quality of cancer diagnosis and treatment in all regions of China.

Certain limitations of this study warrant consideration. First, the utilization of electronic medical records from the hospital as the data source provided a comprehensive and reliable dataset for analysis. However, a limitation of this data source is the absence of socioeconomic characteristics of patients, such as education and household incomes, which are known to be closely related to treatment refusal. The inclusion of these factors could have facilitated a more comprehensive analysis and discussion of the findings. By differentiating health insurance coverage, which primarily encompasses urban employees and residents, it would have been possible to observe variations in health insurance benefits that reflect socioeconomic characteristics to some extent. Second, we extracted the refusal records from the medical record system. There might be missing refusal cases due to incomplete documentation by physicians, which might introduce bias into the results. Furthermore, treatment refusal records extracted by this study included multiple types of treatments, the reasons behind refusal might be multiple and complex, including socioeconomic and medical characteristics of patients and factors of physicians as well as hospitals. The complexity of reasons behind treatment refusal might also introduce bias into the results. Future studies focusing on the refusal cases specifically for targeted therapy would be helpful to address this problem, which requires expansion of the study. The number of patients in a single center is limited, the sample size is too small to enable specific analysis of refusal of targeted therapy. Furthermore, the refusal behavior of quite a high number of patients that we captured for treatment refusal were with unknown reasons. The complexity of reasons of treatment refusal might introduce bias into the analyzes. Another limitation is the single-center design, conducted in a provincial oncology center, which may limit the generalizability of the findings. The inclusion of national cancer centers and cancer hospitals from various provinces would have provided a more representative and statistically robust research sample.

Despite these limitations, the study has two strengths. One is the exhaustive analysis of treatment refusal reasons, which helps to have a comprehensive understand of the reasons behind treatment refusal. The other is the integration of both quantitative and qualitative data analysis. The findings of this study contribute to understanding of the economic factors, insurance coverage, and other variables that affect treatment decision-making among HER2-positive breast cancer patients. The results not only provide a reference for clinical assessment of the reasons for refusing or discontinuing treatment among HER2 breast cancer patients in China, but also bring insights into strengthening the health system in the other developing settings, in order to help cancer patients to overcome the suffering encountered during treatment.

## 5 Conclusion

This study reveals a high rate of treatment refusal among HER2-positive breast cancer patients in southern China, primarily attributed to financial factors. The disparity in health insurance benefits resulted in a heavier economic burden for patients with less comprehensive benefits. Furthermore, the study identified challenges faced by patients seeing quality-assured cancer care in underdeveloped regions in China. By addressing economic barriers, promoting accurate health information and improving cancer care capacity across the country can reduce the rate of treatment refusal.

## Data availability statement

The raw data supporting the conclusions of this article will be made available by the authors, without undue reservation.

## Ethics statement

This study was approved by the Ethics Committee of Fujian Cancer Hospital (K2020-007-01). Patient information was extracted and used by this study with no individual identity. Written informed consent for participation was not required for this study in accordance with the national legislation and the institutional requirements.

## Author contributions

XW: Data curation, Formal analysis, Investigation, Methodology, Visualization, Writing – original draft, Writing – review & editing, Software, Validation. ZL: Data curation, Formal analysis, Methodology, Validation, Conceptualization, Investigation, Resources, Writing – review & editing. QW: Writing – review & editing, Data curation, Investigation. FW: Writing – review & editing, Data curation, Formal analysis, Resources. GZ: Data curation, Writing – review & editing, Project administration, Resources, Validation. JL: Supervision, Writing – review & editing, Conceptualization, Resources. CC: Writing – review & editing, Supervision. JS: Writing – review & editing, Conceptualization, Formal analysis, Funding acquisition, Supervision.
